# Biologics and Non-Biologics Immunosuppressive Treatments for IgA Nephropathy in Both Adults and Children

**DOI:** 10.3390/jcm13092465

**Published:** 2024-04-23

**Authors:** Decimo Silvio Chiarenza, Enrico Eugenio Verrina, Edoardo La Porta, Gianluca Caridi, Gian Marco Ghiggeri, Gabriele Mortari, Francesca Lugani, Andrea Angeletti, Carolina Bigatti

**Affiliations:** Nephrology, Dialysis and Transplantation Unit, IRCCS Istituto Giannina Gaslini, 16147 Genoa, Italy; silvio.chiarenza@yahoo.com (D.S.C.); enricoverrina@gaslini.org (E.E.V.); edoardolaporta@gaslini.org (E.L.P.); gianlucacaridi@gaslini.org (G.C.); gmarcoghiggeri@gaslini.org (G.M.G.); gabrielemortari@gaslini.org (G.M.); francescalugani@gaslini.org (F.L.); carolina.bigatti.cb@gmail.com (C.B.)

**Keywords:** IgA nephropathy, paediatric nephrology, biologics, rituximab, BAFF, APRIL, BAFF/APRIL inhibitors, sibeprenlimab, iptacopan, complement cascade

## Abstract

Immunoglobulin A nephropathy represents the most prevalent cause of glomerulonephritis worldwide and may lead to renal failure in a relevant number of cases in both paediatric and adult subjects. Although their pathogenesis is still largely unclear, evidence of immune abnormalities provides the background for the use of immunosuppressive drugs, such as corticosteroids, calcineurin inhibitors, and antiproliferative and alkylating agents. Unfortunately, these treatments fail to achieve a sustained remission in a significant percentage of affected patients and are burdened by significant toxicities. Recent developments of new biologics, including anti-BAFF/APRIL inhibitors and molecules targeting complement components, offered the opportunity to selectively target immune cell subsets or activation pathways, leading to more effective and safer hypothesis-driven treatments. However, studies testing new biologic agents in IgAN should also consider paediatric populations to address the unique needs of children and close the therapeutic gap between adult and paediatric care.

## 1. Introduction

Immunoglobulin A nephropathy (IgAN) represents the most prevalent cause of glomerulonephritis worldwide, leading to end-stage kidney disease (ESKD) in approximately 20–30% of cases within 25 years after presentation [1,2], and is worsened by the risk of recurrence after kidney transplant in 15–25% of cases [3].

Isolated gross or microhematuria represent the hallmark signs of IgAN. Proteinuria may be present in around half of cases, usually in the non-nephrotic range (<3 g/day), while the development of rapidly progressive glomerulonephritis (RPGN), characterized by the rapid reduction in renal function, is reported to be 2–4% in both adult and paediatric subjects [4,5,6].

The diagnosis of IgAN is based on kidney biopsy, typically showing the expansion and/or proliferation of glomerular mesangium with a dominant or co-dominant mesangial IgA staining [7].

The pathogenesis of IgAN has yet to be completely elucidated and the so-called “4-hit hypothesis” stands as the most widely accepted theory: the cornerstone of the hypothesis is represented by the galactose-deficient IgA1 (Gd-IgA1) production (hit-1) that acts as an antigen for the development of autoantibodies (hit-2). As a consequence, circulating immune complexes composed of Gd-IgA1-autoantibodies (hit-3) deposit in the mesangium (hit-4) [8].

In the last decade, the gut-mucosal-associated lymphoid tissue (MALT), reported to initiate local IgA immune release, was suggested to play a key role in the pathogenesis of IgAN. Several factors, such as the environment, the diet, the microbiome, and genetic predisposition, may affect MALT and contribute to the release of Gd-IgA1 [9]. The type of cells that secrete Gd-IgA1 has yet to be completely clarified, with new data pointing at B cells. Therefore, in last decade, the development of biologic drugs targeting the B-cells’ compartment and components of the complement cascade has been proposed for the treatment of IgAN [10,11].

However, despite a better understanding of the pathogenesis, no specific target therapy has been validated and corticosteroids (CS) still represent the cornerstone of treatment in several adult cases. Of relevance, specific guidelines for the treatment of IgAN recurrence after kidney transplant and IgAN in children are largely missing [12,13].

Therefore, alternative, more selective, and safer therapeutic options are urgently needed. The possible immunosuppressive treatments in IgAN will be the focus of the present review.

## 2. Non-Biologics Treatments 

### 2.1. Corticosteroids

According to the KDIGO guidelines [2] the administration of CS is suggested when proteinuria persists >1 g/day despite optimal supportive care treatment for a minimum of 3 months: dietary sodium restriction, smoking cessation, weight control, and therapy with angiotensin-converting enzyme inhibitors (ACEi) or angiotensin receptor blockers (ARB). More recently, new non-immunosuppressive treatments have been proposed as a supportive therapy with the aim of reducing proteinuria and preventing the decrease in renal function that also occurs in IgAN. In more detail, dapaglifozin, a sodium–glucose cotrasporter-2 (SGLT-2) inhibitor in DAPA-CKD RCT, reduced the risk of CKD progression with an optimal safety profile [14]. On the other hand, sparsentan, a dual endothelin-A receptor and angiotensin II type 1 receptor antagonist [15], was compared with irbesartan in a multicenter, double-blind RCT (PROTECT study) [16] and was found to be more efficient in reducing proteinuria and preserving kidney function at 110 weeks’ follow-up.

CS administration is mainly based on previous studies supporting the efficacy of CS in IgAN. In 1999, Pozzi et al. [17] presented a multicenter RCT where 86 patients with biopsy-proven IgAN were randomly assigned to supportive care therapy or to CS (intravenous methylprednisolone 1 g/day for 3 consecutive days at 1, 3, and 5 months, plus oral prednisone at 0.5 mg/kg on alternate days for 6 months. The main results showed that steroid treatment reduced the risk of a decline in kidney function at 5 and 10 years of follow-up [17,18]. 

Manno et al. [19], in an RCT including 97 biopsy-proven IgAN patients, reported that the combination of CS and ramipril versus ramipril significantly reduced proteinuria and the risk of a renal function decrease at 2 years of follow-up.

More recently, the TESTING trial, a multicenter, double-blind RCT investigating 503 patients, compared placebo with daily methylprednisolone at a dose of 0.4 mg/kg for two months, followed by dose tapering by 4 mg per day for a month. The administration of methylprednisolone correlated with a reduced decline in kidney function at 4 years of follow up [20].

On the other hand, STOP IgAN RCT, which enrolled 162 patients, reported a significant reduction in proteinuria in the CS group compared to the supportive care group (ACEi or ARB), but a similar decline in kidney function [21]. These contrasting results may be justified, at least in part, by the different baseline characteristics of patients among different studies and by the different treatment protocols.

Therefore, based on previous studies and on the KDIGO guidelines, CS should be considered in persistent proteinuria despite optimal supportive therapy, but should be administered with great caution in patients with diabetes, eGFR < 30 mL/min/1.73 m^2^, obesity, latent infections (such as viral hepatitis), or severe osteoporosis; in these patients, CS seems to only offer very short-term benefits. 

In childhood, the efficacy of CS is mostly supported by Japanese studies. In a retrospective study, Yata et al. investigated long-term outcomes (interval between disease onset and end-stage renal disease) in Japanese children and young adults with IgAN who were either receiving CS or not. The study cohort was further divided into two groups based on the year of diagnosis: 1976–1989 and1990–2004. Authors reported an increased improvement in renal survival in patients diagnosed in the period 1990–2004. These results may be partly justified by the development of ACEi in the 1990s [22].

In two different RCT studies enrolling 78 and 80 children with IgAN, prednisolone was found to be effective in preventing declines in renal function [23,24].

Overall, the administration of CS in IgAN, both in adults and paediatrics, should be considered in persistent proteinuria despite optimal supportive therapy. On the other hand, CS only demonstrated short-term benefits when administered in patients with the previously mentioned comorbidities, which are more common in adults (Table 1).

### 2.2. Budesonide

As previously mentioned, the gut mucosal may play a pivotal role in the pathogenesis of IgAN. Nefecon is a targeted-release formulation of the steroid budesonide that acts only on the gut mucosal. The NEFIGAN trial [32], a multicenter, double-blind RCT on 149 patients, compared placebo versus 16 mg/day or 8 mg/day of budesonide. The administration of 16 mg/day of budesonide correlated with a significant reduction in proteinuria. Following these results, a phase 3, multicenter, double-blind, placebo-controlled trial tested the efficacy of Nefecon 16 mg/day administered for 9 months in adults with persistent proteinuria and eGFR > 35 mL/min/1.73 m^2^ [25]. At 15 months of follow-up, the treated group reported a decreased reduction in eGFR and persistent reduction in proteinuria, associated with a satisfactory safety profile (Table 1).

Based on these findings, in 2021, the US Food and Drug Administration (FDA) approved Nefecon for the treatment of patients with IgAN at highrisk of progression to kidney failure with persistent proteinuria despite optimal supportive care and eGFR > 35 mL/min/1.72 m^2^. 

The literature describing the efficacy of budesonide in paediatricIgAN mostly refers to case reports [33,34], and conclusive studies are largely missing.

### 2.3. Cyclophosphamide

Cyclophosphamide is an alkylating agent with chemotherapeutic properties. It forms covalent ligands with DNA, inducing cross-links between DNA strands and subsequent cell death. Cyclophosphamide was largely administrated in several forms of neoplasms and autoimmune diseases [35,36]. According to the KDIGO [2], the administration of cyclophosphamide in IgAN is suggested only in IgAN presenting with RPGN. In line with this, a recent study in 84 patients with IgAN presenting with nephrotic syndrome due to biopsy-proven mesangioproliferative glomerular lesions showed that the combination of cyclophosphamide and CS was more effective in achieving remission than CS alone [PMID: 38046021]. Jia et al. [37], in a retrospective study of 296 patients with chronic kidney disease stage 3 or 4, reported that the administration of CS + cyclophosphamide or CS + mycophenolate mofetil (MMF) significantly increased renal survival and attenuated the rate of renal function decline when compared to supportive treatment. Of note, the authors reported a more effective and safety profile for cyclophosphamide compared to MMF (Table 1).

### 2.4. Mycophenolate Mofetil

MMF is an immunosuppressive agent that inhibits B and T lymphocyte proliferation, potentially reducing the migration of inflammatory cells into glomeruli after antibody deposition. The use of MMF in IgAN remains controversial due to the scarcity of available studies on this topic.

In a recent meta-analysis on nine RCTs (three including paediatric subjects), with a total of 587 patients, the authors reported a similar outcome between MMF and control groups (full-dose steroids and/or supportive treatments) regarding the reduction in proteinuria or decline in renal function [38].

In a recent RCT involving 170 adults with biopsy-proven IgAN in China, MMF correlated with a lower reduction in renal function than the supportive care group [26]. However, in an Italian, two-center, retrospective study, Fontana et al. [39] showed that MMF plus CS and CS alone are similar in inducing complete response (urine protein to creatinine ratio < 0.3 and a less than 20% reduction in eGFR) at 24 months of follow-up (Table 1).

Therefore, based on previous studies, MMF may be considered in cases with persistent proteinuria after a CS cycle, leading to a lower incidence of side effects than CS. The use of MMF as a steroid-sparing agent is mentioned in the KDIGO guidelines, particularly for Chinese patients [2].

### 2.5. Calcineurin Inhibitors

Tacrolimus and cyclosporine, as calcineurin inhibitors, exert immunosuppression by modulating gene transcription in T cells. In more detail, calcineurin inhibitors limit the dephosphorylation of the nuclear factor of activated T cells, thereby hindering the production of interleukin-2 and its receptors. This leads to the direct suppression of T-cell activity, and potentially macrophage activity, coupled with indirect suppressive effects on B-cells [40]. Studies investigating the efficacy of calcineurin inhibitors in the context of IgAN are limited to case series or retrospective experiences.

In a fully comprehensive review of 10 RCTs, evaluating the efficacy of calcineurin inhibitors combined to CS, involving 472 patients, tacrolimus combined with CS was found to be more effective in reducing proteinuria than CS alone or supportive treatments [27] (Table 1).

In a different meta-analysis considering seven RCTs (five with cyclosporin, not included in previous meta-analyses, and two with tacrolimus, included in previous meta-analyses), involving 374 patients, Song et al. reported similar results [28]. 

Therefore, the combination of calcineurin inhibitors with steroids seems to induce a marked reduction in proteinuria compared to steroid monotherapy, indicating a synergistic effect. 

### 2.6. Hydroxychloroquine

Hydroxychloroquine is administered as immunomodulator agent in various autoimmune conditions, including rheumatoid arthritis, systemic lupus erythematosus, and Sjögren’s syndrome. Hydroxychloroquine reduces the activation of T cells and B cells and inhibits the presentation of self-antigens by antigen-presenting cells, thereby preventing the activation of autoreactive T cells. Moreover, it blocks signaling through mucosal and renal Toll-like receptors (TLRs) and decreases the production of pro-inflammatory cytokines and chemokines, thereby mitigating inflammation and tissue damage [41,42] (Table 1). 

Several previous studies, mostly from China, suggested possible efficacy in reducing proteinuria in IgAN [29,43,44,45,46,47]. As the main limitations, all previous studies were characterized by a short follow-up, limiting definitive conclusions on the efficacy of hydroxychloroquine in counteracting the decline in kidney function. 

However, given the safety of hydroxychloroquine and mimicking what already occurs in different immune diseases, the addition of hydroxychloroquine to other therapies in the context of IgAN may be considered a valid supportive strategy.

### 2.7. Leflunomide

Leflunomide reduces the proliferation of T and B cells through the inhibition of di-hydroorotate dehydrogenase, a key enzyme in pyrimidine synthesis. Additionally, it inhibits the activity of tyrosine kinases and NF-kB in T cells [48]. 

Two previous, really extensive meta-analyses [30,31] describing the possible role of leflunomide in IgAN, suggested its efficacy in improving renal function and decreasing proteinuria, mostly when combined with CS. 

Yi J et al. [30] reviewed 35 RCTs and 9 retrospective studies including 1802 patients. The authors reported that the addition of leflunomide to CS or ACEi/ARB may correlate with a better outcome in terms of proteinuria and renal function. 

Lvet al. [31], presented a different meta-analysis of19 RCTs (7 included in previous meta-analyses) including 1153 patients randomly assigned to a treatment group of the combined therapy with leflunomide and CS or CS alone. The combined treatment with leflunomide + CS is more effective than CS alone in reducing proteinuria and preserving renal function.

Furthermore, a more recent retrospective study of 159 patients confirms the efficacy of leflunomide in association with ACEi/ARB in reducing proteinuria and hematuria compared to supportive care alone [46] (Table 1). 

## 3. Biologic Treatments

### 3.1. BAFF/APRIL Inhibitors

As previously reported, the mesangial deposition of Gd-IgA1 characterizes the final step in the pathogenesis of IgAN. Growing evidence suggests that the B-cell-activating factor (BAFF) and A-proliferation-inducing ligand (APRIL) each play fundamental roles in the survival and maintenance of B cells and humoral immunity, and are key mediators between mucosal hyper-responsiveness, B-cell activation, and the consequent overproduction of Gd-IgA1 and its corresponding autoantibodies [49]. In line with this, in a murine model, the increased serum levels of BAFF lead to the development of IgAN, which is prevented in IgA^-/-^mice [50].

Moreover, the serum levels of APRIL are higher in IgAN patients than healthy controls, correlating with the increased levels of circulating Gd-IgA1 and a more severe clinical presentation of the disease [51]. Therefore, BAFF and APRIL emerged as potential and valid therapeutic targets in IgAN.

Atacicept is a human recombinant fusion protein that binds the receptor for both BAFF and APRIL. Atacicept is selective for B-cells, mostly acting on mature B cells and blocking plasma cells and the late stages of B-cell development. Atacicept was recently investigated in the JANUS study [52], a randomized, double-blind, placebo-controlled phase II study, in adult patients with IgAN and persistent proteinuria (UPCR ≥ 1 mg/mg), despite the optimal use of ACEi/ARB. Subjects were randomized into three groups: placebo, and atacicept 25 mg and 75 mg/weekly. Despite the small numbers of patients enrolled (n 16), atacicept was effective in reducing proteinuria and preserving renal function (Table 2). A larger study testing the efficacy of atacicept 150 mg/weekly is in the enrolling phase (ORIGIN phase II study, NCT04716231).

Telitacicept, similar to atacicept, is an APRIL and BAFF inhibitor. In a recent phase II RCT placebo study [53] on 44 adult patients with IgAN and persistent proteinuria (despite the administration of ACEi/ARB), the telitacicept group had a higher reduction in proteinuria than the placebo group (Table 2). A phase III, multicenter, randomized, double-blind, placebo-controlled trial is currently recruiting patients. 

Povetacicept (ALPN-303), a dual BAFF/APRIL antagonist, showed an increased binding affinity and inhibitor activity compared to the previous agents [54]. An open-label study, is currently recruiting patients to evaluate multiple doses of povetacicept in subjects with IgAN and other autoantibody-associated glomerular diseases (RUBY-3, NCT05732402).

Sibeprenlimab is a humanized IgG2 monoclonal antibody that inhibits APRIL. The ENVISION trial [55], a multicenter, double-blind, phase II RCT, evaluated 12 monthly intravenous infusions of sibeprenlimab at doses of 2, 4, or 8 mg/kg versus placebo in 155 patients with IgAN. At 12 months, the sibeprenlimab groups demonstrated a greater reduction in proteinuria compared to placebo (Table 2). A phase III study and an open-label extension study are currently in the recruiting phase (NCT05248646 and NCT05248659).

Zigakibart (BION-1301) is a novel, humanized, monoclonal antibody that blocks APRIL. Preliminary results from a phase 1/2 trial (NCT03945318) suggested that zigakibart is well tolerated, and treatment results in reductions in the serum levels of free APRIL, immunoglobulins, Gd-IgA1, and proteinuria at 52 weeks of follow-up. The global phase 3 BEYOND registrational study (NCT05852938) will evaluate the effect of zigakibart vs. placebo on proteinuria in adults with IgAN.

**Table 2:** Main studies testing biologics in IgAN.

References	Study Design	Follow Up (Months)	Population (n)	Dose	Outcomes
Barratt et al., 2022 [52]	RCT	18	16	Atacicept 25/75 mg sc weekly vs. placebo	Atacicept was effective in reducing serum levels of Gd-IgA1, with significant improvements in proteinuria and renal function compared to the placebo group.
Lv et al., 2022 [53]	RCT	6	44	Telitacicpet 160/240 mg sc weekly vs. placebo	Telitacicept group had lower proteinuria than controls
Mathur et al., 2023 [54]	RCT	12	155	Sibeprenlimab 2/4/8 mg/kg iv monthly vs. placebo	12 months of treatment with sibeprenlimab resulted in a significantly greater decrease in proteinuria than placebo
Zhang et al., 2021 [56]	RCT	6	66	Iptacopan 10/200 mg twice/daily vs. placebo	There was a significant dose-dependent reduction in proteinuria.
Lafayette et al., 2020 [57]	Interventional Study	3	16	Narsopliamb 4 mg/kg iv weekly vs. placebo	Treatment with narsoplimab resulted in a reduction in proteinuria (ranges from 54% to 95% compared to baseline) and the stability of eGFR in high-risk patients
Barratt et al., 2024 [58]	RCT	9	31	Cemdisiran 600 mg every 4 weeks vs. placebo	Significant reduction in proteinuria in treatment group
Bruchfeld et al., 2022 [59]	Interventional Study	3	7	Avacopan 30 mg twice/daily	The improvement in proteinuria observed with Avacopan treatment, along with clinically meaningful improvements in three out of seven patients, suggests the potential efficacy of Avacopan.
Lafayette et al., 2017 [60]	RCT	12	32	Rituximab 1 g twice/monthly	Rituximab failed to significantly improve renal function or proteinuria.
Hartono et al., 2018 [61]	Interventional Study	56	8	Bortezomib 1.3 mg/m^2^ iv	Bortezomib was found to be effective in reducing proteinuria in selected cases of IgA nephropathy. Subjects who responded to bortezomib had an Oxford classification T score of 0 and normal renal function
Tam et al., 2023 [62]	RCT	6	76	Fostamatinib 100/150 mg twice/daily	The study suggested a dose-dependent reduction in proteinuria

RCT: Randomized Controlled Trial; iv: intravenous; eGFR: estimated Glomerular Filtration Ratio; sc: subcutaneous; Gd-IgA1: galactose-deficient IgA1.

### 3.2. Anti-Complement Treatments

The dysregulation of the complement cascade is crucial in the pathogenesis of IgAN [63]. Previous studies have suggested that both the alternative (AP) and the mannose-binding lectin (MBL) pathways may be involved [64,65,66]. Therefore, targeting the complement pathway may represent an attractive therapeutic strategy.

Iptacopan is an oral selective complement inhibitor that specifically binds Factor B and inhibits the AP. A randomized, double blind, phase II study involving 66 patients with biopsy-proven IgAN compared placebo versus iptacopan at four different doses. The administration of 200 mg twice a day was found to be more effective in reducing proteinuria [56].

Following these results, a phase III, multicenter, randomized, double-blind, placebo-controlled, parallel-group study, the APPLAUSE trial (NCT 04578834), evaluating the efficacy and safety of iptacopan, is now enrolling. An interim analysis revealed the superiority of iptacopan compared to placebo in reducing proteinuria at 9 months follow up (Table 2).

Other different molecules targeting Factor B are being tested in ongoing trials (NCT04014335, NCT05797610).

Mannan-binding, lectin-associated serine proteinase 2 (MASP-2) is an effector enzyme that is essential for the activation of the MBL. Narsoplimab, a human monoclonal antibody that inhibits MASP-2, was tested in a phase II study comparing narsoplimab versus CS or ACEi/ARB in 16 patients with IgAN. After 18 weeks of treatment, the narsoplimab group reported a significant reduction in proteinuria compared to controls [57]. However, a subsequent phase III study (ARTEMIS-IgAN, NCT 03608033) failed to demonstrate the efficacy ofnarsoplimab.

Cemdisiran is a subcutaneously administered RNA interference (RNAi) that reduces the transcription of complement component C5. In a phase 2, double-blind, placebo RCT on 31 adults with IgAN, the administration of cemdisiran correlated with a significant reduction in proteinuria [58] (Table 2).

An open-label, phase 2, pilot trial evaluated the efficacy of avacopan, a selective C5a-receptor inhibitor, in seven patients with IgAN: six of the seven patients had an improvement in proteinuria during the treatment period (12 weeks) [59] (Table 2).

An ongoing trial is testing the efficacy of ravulizumab, a humanized monoclonal antibody that inhibits complement protein C5 and is indicated for the treatment of atypical haemolytic uraemic syndrome in patients affected by lupus nephritis and IgAN (NCT04564339).

### 3.3. Rituximab 

The involvement of B cells in IgAN is suggested by the pathological role of anti-Gd-IgA1 antibodies. Rituximab is a chimeric monoclonal-depleting antibody targeting the CD20 antigen expressed on B cells [67]. Rituximab depletes naïve and memory B cells through the induction of apoptosis, antibody-dependent cell-mediated cytotoxicity, phagocytosis, and complement-mediated cytotoxicity [68]. Rituximab has also been associated with the induction of regulatory T cells (Treg), a cell subset that is crucial for the control of autoreactive conventional T cells [69]. Therefore, given the pathological role of anti-Gd-IgA1 antibodies, rituximab represents a treatment option that may limit the release of autoantibodies, selectively targeting the pathogenic mechanism(s) of the disease.

Previous studies describing the administration of rituximab in In IgAN are mostly limited to case series and retrospective studies [70]. However, in a multicenter RCT, Lafayette and colleagues [60] compared rituximab with standard care in 32 IgAN patients, describing similar outcomes in the two groups after 12 months’ follow-up. In line with this, circulating levels of Gg-IgA1 and anti-Gg-IgA1 antibodies were similar to those of the control and treated group, despite the highly effective depletion of B cells (Table 2). 

In contrast to other autoimmune diseases, where rituximab depletes circulating auto-antibodies and improves clinical outcomes [71,72], B cells releasing autoantibodies may mainly derive from the MALT and may be characterized by a limited response to rituximab. Indeed, in patients affected by ulcerative colitis, characterized by the presence of autoantibodies targeting intestinal epithelial cells, rituximab was not effective [73].

Therefore, rituximab does not represent a treatment of choice in IgAN.

### 3.4. Bortezomib

The proteasome, an intercellular protein complex, controls many crucial cell functions: cell-cycle progression, the activation of transcriptional factors, such as nuclear factor-kB (NF-kB), and the production of cytokines and chemokines [74]. The interferons (IFN-gamma or -alfa) induce a switch to a new PS form, the so-called immunoproteasome (iPS), leading to improved peptide presentation to reactive T cells [75]. 

Coppo et al. [76] reported iPS up-regulation in the peripheral blood mononuclear cells of patients with IgAN. 

The proteasome inhibitor Bortezomib was first approved for the treatment of multiple myeloma. It exerts its therapeutic effects by inhibiting the activation of transcriptional factor NF-κB, and by inducing apoptosis in myeloma cells. NF-κB is involved in the pathogenesis of glomerulonephritis, including IgA nephropathy, causing a pro-inflammatory response [77] (Table 2).

In the VELCADE study [61], Bortezomib was tested in eight adult subjects with biopsy-proven IgAN and proteinuria > 1 g/day. Bortezomib was administered in four infusions at the single dose of 1.3 mg/m^2^ and complete remission was reached by only three subjects at 1-year follow-up. The non-responding patients could be due to the increased severity of tubular lesions, according to the Oxford classification [78], suggesting that the efficacy of bortezomib may be limited by the chronicity of the disease.

However, given the small sample size and the lack of a control group, more studies are needed to test the efficacy of bortezomib in IgAN. An ongoing interventional study (NCT05383547) is testing bortezomib in glomerular disease (such as IgAN, membranous nephropathy, and focal segmental glomerulosclerosis), characterized by proteinuria > 1.5 g/day.

### 3.5. Fostamatinib

Fostamatinib is a small-molecule inhibitor that targets spleen tyrosine kinase, which plays a crucial role in B-cell receptor signaling. It is used in the treatment of certain autoimmune conditions, such as immune thrombocytopenia and rheumatoid arthritis, by modulating B-cell function and reducing inflammatory responses [79].

The efficacy of fostamatinib in IgAN was recently tested in a double-blind, randomized, placebo-controlled, phase 2 trial with 76 patients. Although no significant reduction in proteinuria with fostamatinib was detected overall, there was a trend for a dose-dependent reduction in median proteinuria [62] (Table 2). However, further study may be warranted.

## 4. Conclusions

The limited efficacy and toxicities associated with the use of CS have compelled the search for alternative treatment strategies. Non-biologic agents, such as MMF, hydroxychloroquine, and budesonide, may be used as CS-sparing agents or as an additional therapy in those patients who remain at high risk of progression despite receiving optimal supportive care. Amongst biologics, BAFF/APRIL inhibitors and molecules targeting complement components recently emerged as valid strategies to effectively reduce the rate of the decrease in renal function in patients with IgAN. Further studies proving the effective/safety profile of such treatments are ongoing and largely needed. 

On the other hand, there is often a therapeutic gap between the treatments available for adults and those for children. This can result in limited treatment options for paediatric patients, leaving them with suboptimal care or facing the risk of the off-label use of medications. Upcoming studies testing new biologic agents in IgAN should also consider paediatric populations to address the unique needs of children and close the therapeutical gap between adult and paediatric care.

## Figures and Tables

**Table 1:** The main studies testing non-biologic immunosuppressive treatments in IgAN.

References	Study Design	Follow Up (Years)	Population (*n*)	Dose	Outcomes
Pozzi et al., 1999 and 2004 [17,18]	RCT	5, 10	86	Methylprednisolone 1 g/day for 3 consecutive days for 1, 3, and 5 months plus oral prednisone 0.5 mg/kg on alternate days for 6 months vs. placebo	Steroid treatment reduced the risk of a decline in kidney function at 5 and 10 years of follow-up.
Manno et al., 2009 [19]	RCT	8	97	Prednisone 1 mg/kg/day for 2 months and then tapered by 0.2 mg/kg/day every month, plus ramipril vs. ramipril	Combination of steroids and ramipril may provide additional benefits compared with ramipril alone in preventing the progression of renal disease.
Lv et al., 2022 [20]	RCT	Mean 4.2	503	Methylprednisolone 0.4 mg/kg/day for 2 months, and then tapered by 0.4 mg/kg/day every month vs. placebo	Steroids significantly reduced the risk of kidney function decline, kidney failure, or death due to kidney disease.
Rauen et al., 2020 [21]	RCT	3	162	Methylprednisolone 1 g/day for 3 consecutive days at 1, 3, and 5 months plus oral prednisone 0.5 mg/kg on alternate days for 6 months plus RAASi vs. RAASi alone	The addition of steroids to intensive supportive care in patients with high-risk IgA nephropathy did not significantly improve the outcome
Yoshikawa et al., 1999 [23]	RCT	2	78 Paediatric subjects	Prednisolone 2 mg/kg (max. 80) for 2 months, then 1.5 mg/kg for 1 month then 1 mg/kg for 21 months plus azathioprine 2 mg/kg/day, plus heparin and warfarin, plus dipyridamole vs. heparinandwarfarin, plus dipyridamole	Treatment of children with severe IgAN with prednisolone, azathioprine, heparin-warfarin, and dipyridamole prevents immunologic renal injury and increases in sclerosed glomeruli, and may lead to remission
Lafayette et al., 2023 [25]	RCT	2	364	Nefecon 16 mg/day vs. placebo	Nefecon provided a clinically relevant reduction in eGFR decline and a durable reduction in proteinuria.
Hou et al., 2023 [26]	RCT	3	170	Mycophenolate mofetil 1.5 g/day for 12 months, and then tapered to 0.75–1 g/day for at least 6 months plus steroids vs. steroids	The addition of mycophenolate mofetil to steroids significantly reduced the risk of doubling of serum creatinine, ESKD, or death due to kidney or cardiovascular causes
Kim et al., 2013 [27]	RCT	0.3	40	Tacrolimus twice daily (serum levels at 5–10 ng/mL) vs. placebo	Tacrolimus reduced proteinuria effectively and rapidly in IgA nephropathy with mild to moderate proteinuria
Xu et al., 2014 [28]	RCT	1	96	Cyclosporine 3 mg/kg/day per 3 months, then 2 mg/kg/ for 9 months plus prednisolone 0.6–0.8 mg/kg/day for 6–8 weeks vs. prednisolone 1 mg/kg/day for 8–12 weeks	The combination of Cyclosporine and steroids was more effective in reducing proteinuria and elevating plasma albumin
Liu et al., 2019 [29]	RCT	0.5	60	Hydroxychloroquine 0.2/0.1 g twice daily or 0.1 g thrice daily according to eGFR plus RAASi vs. placebo plus RAASi	Hydroxychloroquine in addition to optimized RAASi significantly reduced proteinuria
Min et al., 2017 [30]	RCT	1	85	Leflunomide 40 mg/day for 3 days, then 20 mg/day for 12 months plus prednisone 0.8 mg/kg/day (max. 40) for 4–6 weeks, then tapered vs. prednisone 1 mg/kg/day for 8–12 weeks	Leflunomide combined with low-dose CS seems to be at least as effective as full-dose CS for the treatment of progressive IgAN, and is associated with both a greater reduction in proteinuria and fewer severe adverse events
Ni et al., 2021 [31]	RCT	2	108	Leflunomide 40 mg/day for 3 days, then 20 mg/day for 12 months plus prednisone 0.5–0.8 mg/kg/day (max. 40) for 8–12 weeks, then tapered vs. prednisone 1 mg/kg/day for 8–12 weeks	The efficacy and safety of leflunomide plus low-dose-prednisone and prednisone alone in the treatment of progressive IgAN are comparable

ESKD: end-stage kidney disease RAASi: renin–angiotensin–aldosterone system inhibition.

## Data Availability

Not applicable.
